# MRI feature tracking strain profiles distinguish patients with left ventricular systolic and diastolic dysfunction with and without clinical heart failure

**DOI:** 10.1186/1532-429X-18-S1-O79

**Published:** 2016-01-27

**Authors:** Naila Choudhary, Lynette J Duncanson, Javed Butler, Nathaniel Reichek, Timothy Vittorio, Alistair Young, Jie J Cao

**Affiliations:** 1 University of Florida College of Medicine, Jacksonville, FL USA; 2 grid.36425.360000000122169681SUNY Stony Brook University, Stony Brook, NY USA; 3 grid.416387.fSt. Francis Hospital - The Heart Center, Roslyn, NY USA; 4 grid.9654.e0000000403723343University of Auckland, Auckland, New Zealand

## Background

Characterizing myocardial mechanical properties is valuable in understanding cardiomyopathy with and without clinical heart failure (HF). We sought to examine the incremental value of strain assessment in patients with left ventricular systolic dysfunction without HF (LVSD), HF with reduced ejection fraction (HFrEF), LV diastolic dysfunction without HF (LVDD) and HF with preserved EF (HFpEF) during routine cardiac MR (CMR) evaluation.

## Methods

All study participants were enrolled prospectively to undergo CMR in a 1.5 T scanner. HF was diagnosed with clinical manifestations and elevated BNP (>400 pg/ml) or NT-BNP (> 900 pg/ml). Patients with myocardial infarction and severe mitral regurgitation were excluded. LVSD was defined as having LVEF < 50%. LVDD was determined by echocardiographic criteria or by elevated LV filling pressure assessed during cardiac catheterization. Myocardial circumferential strain (CS) and strain rate (CSR) were taken as the average of basal, mid and apical CS and CSR of the CMR cine images using feature tracking (CIM 3 software, Auckland, New Zealand). The longitudinal strain (LS) and strain rate (LSR) were analyzed in 4-chamber view.

## Results

Mean LVEF was 26 ± 10% for HFrEF (N = 16) and 35 ± 8% for LVSD (N = 14). The average CS was lowest in HFrEF (-8 ± 3%) followed by LVSD (-9 ± 3%) compared to normal (-17 ± 2%) (N = 11) (p < 0.001). In contrast, CS was largely unaltered in LVDD (-15 ± 4%) (N = 14) and in HFpEF (-18 ± 3%) (N = 10). While HFrEF demonstrated lowest LS (-6 ± 3%) which was followed by LVSD (-9 ± 2) and HFpEF (-9 ± 3%) when compared to normal (-14 ± 2%) (p < 0.001), LVDD did not show significant LS reduction (-14 ± 2%). Findings of CSR and LSR were similar to CS and LS, respectively (both p < 0.001). The early diastolic relaxation rate in the circumferential direction dropped by ~50% in HFrEF and LVSD but it was largely unchanged in HFpEF and LVDD. However, LVDD demonstrated significantly enhanced late diastolic relaxation rate (45 ± 15%) compared to normal (22 ± 9%) (p < 0.001) whereas other groups did not.

## Conclusions

In contrast to reduced CS and LS in HFrEF and LVSD, HFpEF is characterized by preserved CS but markedly reduced LS. While LVDD has preserved CS and LS it is associated with heightened late diastolic relaxation rate. Identifying these distinctive myocardial mechanical features can be an important addition to CMR evaluation of cardiac function and tissue characteristics.

